# Magnetic biomaterials and nano-instructive tools as mediators of tendon mechanotransduction

**DOI:** 10.1039/c9na00615j

**Published:** 2019-12-05

**Authors:** Ana M. Matos, Ana I. Gonçalves, Alicia J. El Haj, Manuela E. Gomes

**Affiliations:** 3B's Research Group, I3Bs – Research Institute on Biomaterials, Biodegradables and Biomimetics, University of Minho, Headquarters of the European Institute of Excellence on Tissue Engineering and Regenerative Medicine Avepark – Zona Industrial da Gandra, 4805-017 Barco Guimarães Portugal ana.goncalves@i3bs.uminho.pt megomes@i3bs.uminho.pt; ICVS/3B's – PT Government Associate Laboratory Braga/Guimarães Portugal; The Discoveries Centre for Regenerative and Precision Medicine, Headquarters at the University of Minho Avepark, 4805-017 Barco Guimarães Portugal; Healthcare Technologies Institute, Birmingham University B15 2TT Birmingham UK

## Abstract

Tendon tissues connect muscle to bone allowing the transmission of forces resulting in joint movement. Tendon injuries are prevalent in society and the impact on public health is of utmost concern. Thus, clinical options for tendon treatments are in demand, and tissue engineering aims to provide reliable and successful long-term regenerative solutions. Moreover, the possibility of regulating cell fate by triggering intracellular pathways is a current challenge in regenerative medicine. In the last decade, the use of magnetic nanoparticles as nano-instructive tools has led to great advances in diagnostics and therapeutics. Recent advances using magnetic nanomaterials for regenerative medicine applications include the incorporation of magnetic biomaterials within 3D scaffolds resulting in mechanoresponsive systems with unprecedented properties and the use of nanomagnetic actuators to control cell signaling. Mechano-responsive scaffolds and nanomagnetic systems can act as mechanostimulation platforms to apply forces directly to single cells and multicellular biological tissues. As transmitters of forces in a localized manner, the approaches enable the downstream activation of key tenogenic signaling pathways. In this minireview, we provide a brief outlook on the tenogenic signaling pathways which are most associated with the conversion of mechanical input into biochemical signals, the novel bio-magnetic approaches which can activate these pathways, and the efforts to translate magnetic biomaterials into regenerative platforms for tendon repair.

## Abstract 2

This review identifies the latest advances and potential of magnetically actuated biomaterials within the scope of tendon tissue engineering.

## Graphical abstract

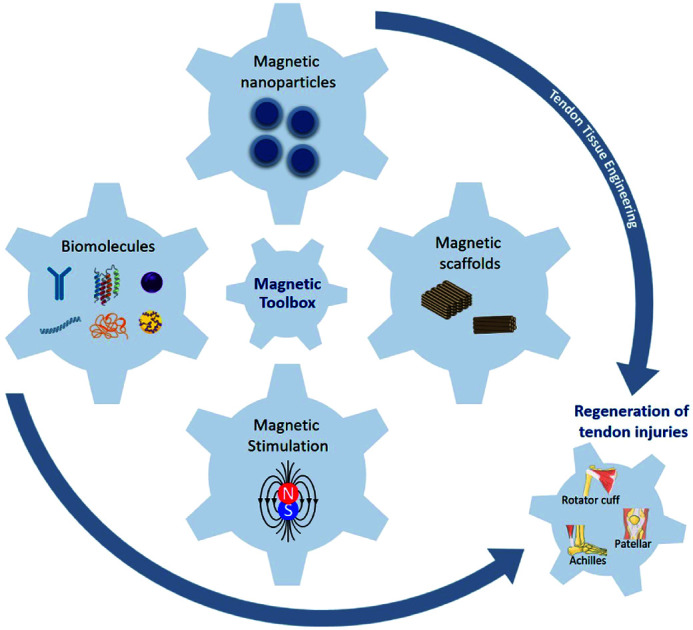

## 1. Introduction

Tendons are transmitters of forces generated by muscle to the bone. Tendons are one of the tissues exposed to the most extreme mechanical forces in the body.^1^ The Achilles tendon is the thickest tendon in the human body^2^ and it can receive a load stress 3.9 times the body weight during walking and 7.7 times the body weight during running.^3^ The frequent exposure of these tissues to high mechanical stresses leads to a high incidence of damage in tendons. The overuse of tendons is a significant problem in individuals who perform repetitive activities, both in sports and at work,^4^ and it is estimated that 30–50% of all injuries related to sports medicine involve tendinopathy.^5,6^ In fact, this musculoskeletal disease has a significant impact on health care system expenditure making the investigation of molecular mechanisms involved in tendon repair essential to develop novel treatment therapies.

Presently, tissue engineering is an emergent field that could become a real therapeutic option in the treatment of tendon injuries. As transmitters of forces and as mechanoresponsive tissues, the delivery of stimuli is of utmost importance in tissue engineering approaches aimed at tendon regeneration. Moreover, cells within tissues perceive a complex microenvironment in terms of extracellular signals, chemical compounds, and metabolic precursors and intermediates, or even physical properties of their surroundings.^7^ Mechanobiology has revealed that such environmental cues and cellular mechanotransduction can be pivotal in a variety of responses, such as apoptosis, division, migration, and differentiation. Thus, given the recognition of the importance of biomechanical cues for mechanotransduction events, biomechano-responsive materials have emerged as promising platforms to realize biomedical functions.^8,9^ In the tendon tissue engineering field, the appropriate combination of teno-inductive cues such as appropriate cells, stimuli-responsive biomaterials, and mechanical stimuli is of key importance to boost tenogenic differentiation.^10–14^ Overall, biomechanical stimuli generated by either endogenous forces (tensile, compressive, and shear forces) or exogenous forces (ultrasound and magnetic forces), can be exploited as triggers for mechanoresponsive materials to be interfaced with biological systems.^8^

Magnetically responsive biomaterials and magnetotherapy are potential actuators that may enable cell stimulation both *in vitro* and *in vivo*, due to the feasibility of remote non-invasive actuation, post transplantation. Additionally, magnetic forces induced by a magnetic field can remotely and noninvasively activate the magneto-responsive components embedded in the scaffold matrix or attached to the cells. In this review, we briefly overview the tendon structure and the importance of mechanical stimulation to maintain tendon homeostasis, summarizing the signaling cascades involved in mechanotransduction. Finally, some insights are given into tackling tendon regeneration through magnetically assisted tissue engineering tools and magnetic biomaterials serving as mediators of mechanotransduction.

### 1.1. Tendon structure and composition

The tendon presents highly intricately organized structure that supports forces with large magnitudes between the muscles and bones during daily activities. This structure depends on the interaction between local cell types and regulation of extracellular matrix (ECM) remodeling.^15,16^ The mechanical properties of tendon tissue derive from type I collagen fibers that are arranged in dense parallel arrays.^17^ Tropocollagen is a triple-helix type I collagen molecule which is synthesized by tendon fibroblasts or tenocytes.^18^ A myofibril is five tropocollagen molecules stacked in a quarter-stage array^17^ and, in turn, neighboring microfibrils interdigitate and form a fibril which is the smallest tendon structural unit with a 10–500 nm diameter depending on species, age and location.^19^ Fibers are composed of collagen fibrils having a diameter between 3 and 7 μm, which are bound by the *endotenon*, a thin layer that contains blood vessels, lymphatics and nerves.^19^ An ensemble of fibers forms fascicles that are bundled together through a fascicular membrane designated *epitenon*,^17^ which is a fine, loose connective tissue containing vascular, lymphatic and nerve supply to the tendon.^20^ The fascicles, closely packed and arranged in parallel, form the tendon. Surrounding the *epitenon*, there is a layer of loose areolar connective tissue consisting of type I and type III collagen fibrils organized in a perpendicular direction called the *paratenon*.^18^ The set of these two layers that surround the tendon is called the *peritendon*, and it has the function of reducing friction with adjacent tissues.

The conservation of the highly organized structure of this tissue is carried out by cells and it is crucial for maintaining mechanical properties and preventing injury. The three main cellular types present in tendons are tenoblasts, tenocytes and tendon stem/progenitor cells (TPCs/TSPCs). Tenoblasts can be stimulated to differentiate into tenocytes in response to various stimuli, such as exercise and trauma, in order to induce proliferation and matrix remodeling.^21,22^ These fibroblast-like cells are capable of producing collagen type I and ECM components and can be found between collagen fibers and the *endotenon*.^15,21,23^ These cells are organized in linear arrays along the long axis of the tendon and interspersed between the collagen fibers.^24^ TSPCs are another type of tendon cell, recently discovered, which exhibit regenerative capacity and have an important role in tendon maintenance and repair.^16,25,26^ Directly surrounding the cells is the pericellular specialized matrix^27^ that may have an important role in mechanobiological mechanisms since it is composed of type VI collagen, elastin and fibrillin-1 which maintain the structural and biomechanical integrity of the tendon.^27^

In addition to collagen, the ECM is composed of other components, such as elastin, glycoproteins, proteoglycans and other molecules like collagen oligomeric matrix protein (COMP) and lubrican.^28^ Several of these proteins have the ability to regulate fibrillogenesis in terms of fibril diameter, alignment and stability.^29–31^ Elastin represents 1–2% of the tendon dry weight^32^ and provides flexibility for distension during unidirectional elongation.^15^ Furthermore, this protein has the ability to recover the configuration of fibers after mechanical loading.^21^ Among the most important glycoproteins are tenascin C and fibronectin, which enhance mechanical stability, allowing tendons to return to their prestretched lengths after physiological loading.^33^ Thrombospondin-4 (TSP4) is a glycoprotein abundant in mature tenocytes and is associated with fibrillar structures due to regulation of collagen assembly, organization, and ECM remodeling.^34,35^ Small leucine-rich proteoglycans (SLRPs) are abundant proteoglycans present in the ECM and function to regulate collagen fibril self-assembly;^36^ fibromodulin is one of the most expressed in tendons and crucial for the organization of the collagen fibril structure.^37^ Furthermore, other proteins are important for tendon development such as tenomodulin (TNMD), highly expressed in developing and mature tendons being a key marker for differentiated tenocytes.^38^ Also, TNMD was recently reported as a mechanosensitive gene required for proper tendon function since its expression in human TSPCs rapidly decreases in static cultures but is restored upon axial stretching, indicating the mechanosensitivity of the *Tnmd* gene.^39^

## 2. Tendon response to mechanical stimuli

As discussed above, tendon mechanical properties derive largely from type I collagen fibers which are arranged in dense parallel arrays. This arrangement results in a resilient tissue with high tensile stiffness in the direction of fiber orientation.^40^ Moreover, tendons present high mechanical strength and good flexibility and viscoelasticity which make this tissue more deformable at low strain rates and less deformable at high strain rates. However, at low strain rates tendons absorb more mechanical energy but are less effective in transmitting loads. At higher strain rates, tendons become stiffer and more effective in transmitting high muscular loads to bones.^19,33^ In addition to this mechanical behavior, each tendon has different mechanical properties depending on the function it performs in the body. For example, the human patellar tendon exhibits a Young's modulus of 660 ± 226 MPa (ref. 41) whereas the Achilles tendon has a Young's modulus around 1671.02 ± 277.5 MPa^42^ as it sustains the body weight and has a major impact on postural orientation. Mechanical loading is important for development and homeostasis maintenance of the tendon, and its physiological values are dependent on the tendon's function, gender, age, species and location.^43^ This condition is maintained with a regulated balance of the ECM between matrix metalloproteinases (MMPs) and tissue inhibitors of metalloproteinases (TIMPs).^44^

Tendons are mechanosensitive tissues that respond to load in an adaptive manner, which means that the tendon alters its biological structure and its mechanical behavior in response to various mechanical stimuli^33^ resulting in changes in growth factor or cytokine expression.^17^ In this context, tenocyte sensitivity to their environment has been well studied *in vivo* and *in vitro*.^45–49^ There are four mechanisms by which cells respond to mechanical forces such as activation of ion channels, release of ATP, changes in cytoplasmic filament organization and composition, alteration of protein expression and secretion of MMPs.^50^ It is suggested that mechanical loading induces biochemical changes which, in turn, increase repair and remodeling by tenocyte activity stimulation. However, overloading of the tissue leads to injuries, altering its structure and mechanical properties, and increases the production of inflammatory mediators.^51,52^ On the other hand, insufficient mechanical loading leads to changes in the cellular shape, number of cells and collagen fiber alignment, which culminate in degeneration of the tissue. Moreover, the absence of mechanical forces leads to tissue atrophy and, consequently, loss of its weight, stiffness, and the capacity to support tensile forces without being damaged.^19,33^

### 2.1. Tendon mechanotransduction and signaling pathways

The coordination of cell growth and proliferation with the production of the ECM is responsible for tissue homeostasis. This is achieved by cell signaling, in which a cell secretes a cytokine acting on that same cell (autocrine activity) or on another cell (paracrine activity), which regulates tissue remodeling.^53^ Mechanotransduction is the ability of cells to respond to mechanical stimuli through biochemical signals.^50^ These stimuli are transduced by cells to stimulate biochemical pathways and effective cellular processes such as differentiation, proliferation, tissue development and skeletal maintenance.^54^

Cells can perceive external mechanical stimuli through integrins, cadherins, catenins, stretch-activated ion-channels, and growth factor receptors. Integrins are transmembrane heterodimer proteins composed of α and β subunits that physically couple the ECM to the cytoskeleton through linker proteins, conveying forces between the inside and outside of the cell.^50^

The manipulation of integrin attached magnetic particles and internalized particles has been shown to induce intracellular calcium signalling in human osteoblasts^55^ and in hMSCs.^56,57^ Particularly in tendons, collagen I-binding integrins, α1, α2 and α11, were strongly upregulated and the integrin downstream kinases p38 and ERK1/2 were activated in mechanically loaded TSPCs.^58^

**Fig. 1:**
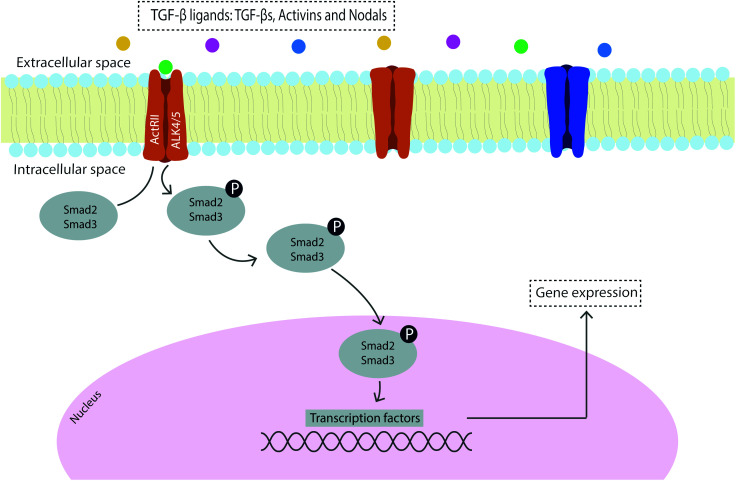
Schematic representation of the TGF-β/Smad2/3 signaling cascade.

Signal transduction can occur through several mechanisms and signaling pathways^59^ with the main growth factors involved in vertebrate tendon development being transforming growth factor (TGF)-β and fibroblast growth factor (FGF), which are transduced *via* SMAD2/3 and ERK/MAPK cascades, respectively.^60^ Moreover, the bone morphogenetic protein (BMP) related members of the TGF-β family are elevated in early tendon healing processes and are transduced *via* the BMP/SMAD1/5/8 signaling pathway.^61,62^ The family of TGF-β ligands includes TGF-βs, activins, NODAL, bone morphogenetic proteins (BMPs), growth and differentiation factors (GDFs) and the anti-Müllerian hormone (AMH),^63^ and the mechanism of signaling constitutes a cascade of phosphorylation events to transduce the signal to the nucleus and consequently regulate gene expression (Fig. 1). The sequential cascade of phosphorylation is initiated by binding of ligands and activation of type I and type II receptor serine/threonine kinases on the cell surface, propagating the signal through phosphorylation of SMAD transcription factors.^62^

TGF-βs are therefore major regulators of differentiation, proliferation and ECM production in connective tissues which act as mediators of tendon development, differentiation and homeostasis.^59,60^ More specifically, TGF-β is present in the tendon ECM and is released in response to exercise and strain to regulate the synthesis of collagen, acting as a mechanical transducer of mechanical force into TGF-β mediated biochemical signals.^64^ Furthermore, TGF-β has been found to have an important role in angiogenesis, gliding surface restoration and modulation of adhesion formation which evidence the role of this well-known tendon healing regulator in improving tendon repair.^65^

Mechanosensory molecules downstream of mechanical forces are the transcription factors basic helix–loop–helix transcription factor, scleraxis (Scx), the homeobox protein Mohawk (Mkx), and the zinc finger transcription factor early growth response 1 (Egr1). Scx is an early tendon specific marker, associated with ECM organization and development of functional *de novo* tissue.^40,66^ Unlike Scx, Mkx, and Egr1 are not specific to tendons, but each of the three alone is able to induce tenogenesis in stem cells.^40,67^ Mkx appears to regulate collagen fibril growth during tendon development since when it is absent there is a reduction in collagen gene expression during the fetal stage and a decrease in the amount of type I collagen in mature tendons.^23,68^ Egr1 was shown to promote tenogenesis in stem cells and improve tendon healing and repair in animal models of tendon injury,^69,70^ and it may play a role in regulating tendon ECM formation by controlling collagen type I deposition and fibrillogenesis.^71^

## 3. Tackling tendon regeneration through magnetically assisted tissue engineering tools

Mechanical loads generated by gravity and locomotion stimulate tendon remodeling to maintain the optimal mechanical performance of the tissue. Magnetic mechano-actuation is an interesting approach to remotely deliver mechanical stimulation directly to individual cells, as magnetic nanoparticles (MNPs) attached to the cell membrane can be manipulated using an oscillating gradient of external magnetic fields thus applying forces in the pN range to the particles and activating the receptors.^72,73^ The magnetic fields applied in these strategies can be either static magnetic fields (SMFs) or electromagnetic fields (EMFs): the SMFs are constant fields that exert an attractive force on metallic objects, so magnets are commonly used for this purpose; the EMFs result from a combination of electric and magnetic fields, that is, the magnetic field is produced by the movement of electrically charged particles. The most commonly used EMF is a pulsed electromagnetic field (PEMF) since it is FDA approved, non-invasive,^74^ can be applied directly to the treatment site, and also enables varying signal configurations to modulate the cells' response at the molecular level, acting as a mediator of inflammation in the treatment of tendinopathic disorders or to prevent post-operative re-tears.^75,76^

Commercial devices are available which are capable of generating magnetic fields with different patterns^11,77,78^ and custom-designed systems can be developed according to the purpose of the application in the field.^79–83^ Combination strategies which include magnetic stimulation and magnetic biomaterials may boost cell signal transduction by means of remote activation of mechanotransduction pathways.

### 3.1. Magnetic biomaterials

Over the past few years, there has been considerable interest in magnetic biomaterials in biomedicine, in particular because the properties of these materials can be controlled in a remote fashion enabling non-invasive (noncontact) forms of actuation. The use of MNPs, especially superparamagnetic iron oxide nanoparticles (SPIONs), in biomedical and tissue engineering applications has exceeded expectations, mainly because of their superparamagnetic behavior which makes them desirable as a magnetic-targeting tool for medical applications. SPIONs are composed of a magnetic core of magnetite (Fe_3_O_4_) or maghemite (Fe_2_O_3_) and are often polymer coated^84^ to improve their biocompatibility and structural and colloidal stability, while providing functional groups for bioactive molecule and/or ligand conjugation for targeting cells or tissues.^85^ Polymeric composites incorporating superparamagnetic iron oxide particles is one approach for remotely actuate biomaterials^8^ using magnetic fields as exogenous mechanical triggers to exert forces over seeded layers of cells. Thus, the incorporation of MNPs within a 3D scaffold or hydrogel and/or the association of MNPs with stem cells result in magnetically responsive systems suitable for tissue engineering applications.^85–87^

Sapir-Lekhovitser and co-workers hypothesized that magnetic fields coupled with magnetizable nanoparticles embedded within 3D scaffold structures remotely create transient physical forces that can be transferrable to cells present in close proximity to the nanoparticles.^87^ It was estimated that magnetic fields as low as 1.5 mT on alginate-based magnetic scaffolds incorporating MNPs applied forces on endothelial cells of about 1 pN,^87^ which is in good agreement with the reported threshold of 0.2 pN required to induce mechanotransduction effects at the cellular level.^88,89^ In fact, the application of time-varying external magnetic fields applies either a translational force (due to the attraction of MNPs along the magnetic field gradient) or a combination of translational and torque forces, which are transmitted directly to the cell membrane or the cytoskeleton, and can be varied in three dimensions within a scaffold.^89^

Our group has been keen on developing magnetic systems for tendon tissue engineering purposes. The effect of an externally applied magnetic field on tenogenic differentiation of human adipose stem cells (hASCs) was assessed by culturing cells on a magnetic scaffold. An aligned fibrous structure of starch with poly(ε-caprolactone) (SPCL) incorporating iron oxide MNPs was fabricated by 3D printing technology. The results showed that the effect of the aligned magnetic scaffolds combined with magnetic stimulation promotes tenogenic differentiation of hASCs suggesting the potential of the developed system to activate mechanotransduction pathways that are responsible for tenogenic commitment.^11^ Moreover, and to understand the effect of magnetically actuated biomaterials in modulating the inflammatory process of tendons, a magnetically actuated SPCL membrane was produced and implanted subcutaneously in rats.^77^ The results showed that the magnetic membranes under PEMF stimulation maintain metabolic activity, proliferation and reactive oxygen species production by hASCs as well as preventing the formation of scar tissue by decreasing the presence of profibrotic inflammatory cells surrounding the explanted biomaterials.^77^ More recently, Tomás *et al.* used the setup proposed in a previous study^90^ to produce yarns of continuous and aligned electrospun threads of PCL and cellulose nanocrystals (CNCs) coated with iron oxide magnetic nanoparticles, resulting in magnetically responsive fibrous scaffolds.^91^ Cell studies revealed that magneto-mechanical stimulation of hASCs promotes higher degrees of cell cytoskeleton anisotropic organization, increased expression of tendon-related markers, and an anti-inflammatory gene profile. This work suggests a synergistic effect of nanotopography and magneto-mechanical actuation on the tenogenic commitment.^91^

Magnetically responsive hydrogels of methacrylated chondroitin sulfate (MA-CS) coated with iron-based MNPs^92^ and tropoelastin magnetic sponge-like hydrogels^78^ were also developed as 3D carriers of magnetic fields to the cells modulating the biochemical, physical and mechanical properties of the surrounding environment. By the application of EMF stimulation, it was possible to control the intrinsic properties of the constructs. Moreover, EMF stimulation of human tendon-derived cells and osteogenically differentiated hASCs was capable of modulating the cellular response of both cellular types.^92^

In summary, magnetic materials have the potential to enhance cell behavior promoting the activation of signaling pathways involved in tendon development and homeostasis by delivery of mechanical cues through remote generation of an external magnetic field.

### 3.2. Remote activation of mechanotransduction pathways

**Fig. 2:**
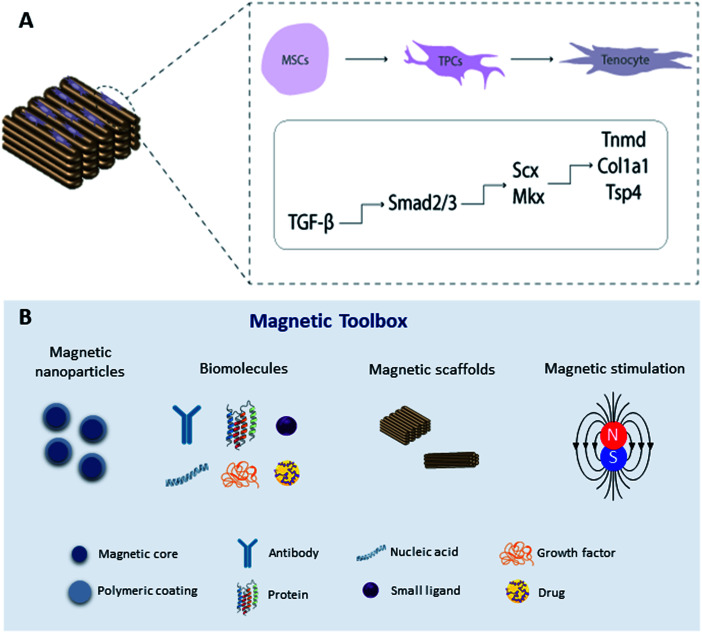
(A) Schematic representation of transcriptional regulation of tendon specific markers by activation of the TGF-β/Smad2/3 signaling pathway on 3D magnetic constructs; (B) magnetic toolbox: magnetic nanoparticles can be functionalized with biomolecules responsible for activating signaling cascades through remote magnetic stimulation. Legend: mesenchymal stem cells (MSCs), tendon progenitor cells (TPCs).

An alternative approach is to magnetically tag specific receptors on the cells with magnetic nanoparticles^93,94^ which have been functionalized with specific receptor targets which can be mechano-activated *via* remote magnetic fields. Magnetic mechano-activation remotely delivers mechanical stimuli directly to cells which are transmitted through activation of mechanically sensitive receptors available on the cell membrane. This activation initiates signaling pathways enabling cells to respond to mechanical cues in the environment through biochemical signals that dictate downstream cellular responses in many cases leading to differentiation. The use of MNPs, magnetic biomaterials, and magnetic fields is increasingly becoming a hot topic in regenerative medicine to regulate cell fate by manipulating mechanotransduction (Fig. 2).

Previous studies have explored the use of MNPs targeting PDGF,^95^ TREK-1,^96–98^ Wnt,^99,100^ and ActRIIA^101^ as actuators of signaling pathways in human mesenchymal stem cells (hMSCs) for tissue engineering in *in vitro* and *in vivo* approaches. To target the mechano-responsive ion channel TREK-1, hMSCs were labeled with TREK-1 functionalized MNPs under magnetic stimulation. The results demonstrated that this approach can directly stimulate cells and selectively activate the mechanosensitive ion channel TREK-1 promoting osteogenic differentiation of hMSCs.^57,96–98^ Magnetic mechano-activation was also explored to induce tenogenic differentiation of hASCs which were labelled with MNPs functionalized with anti-activin receptor type IIA antibody to remotely activate the TGF-β/Smad2/3 signaling pathway. The results showed phosphorylation of Smad2/3 proteins in MNPs–ActRIIA tagged hASCs potentiating the commitment into the tenogenic lineage *via* TGF-β/Smad2/3.^101^ These findings emphasize the role of magnetic actuation in the activation of cell receptors and triggering of signaling cascades, giving rise to a wide range of opportunities to remotely control stem cell commitment. Recent work has translated this approach to large animal models for bone regeneration.^96^ However, more investigation is needed to fully understand the effect of this approach on tendon regeneration, including studies to explore different target receptors, combining this approach with magnetic biomaterials, and optimizing instrumentation for delivering magnetic stimuli.

## 4. Conclusions

It is becoming increasingly clear that understanding the basis of mechanotransduction plays an important role in developing successful tissue engineering and regenerative medicine therapies. Scaffolding systems can serve as valuable platforms for studying cell mechanotransduction in three-dimensional environments that recap the native cellular niche. Using magnetic biomaterials and magnetic mechano-actuation, we can further explore novel approaches and concepts to target key challenges in the field. Hence, unexplored areas such as triggering mechanotransduction using the most recent nanotechnology tools for tendon tissue engineering present major opportunities for research on advancing regenerative solutions. Magnetic actuation of cells and cellular constructs and promotion of mechanotransduction shed light on the remote activation of intracellular responses and tissue formation in cell therapies. Furthermore, combining magnetic biomaterials and remote magnetic mechano-activation of signaling pathways is a potential regenerative magnetic toolbox as yet hardly explored in tendon tissue engineering, which might harness new biomedical possibilities in the regeneration of tendons. Concomitantly, the delivery of functional stimuli *in vivo* and the ultimate translation of this technology constitutes an important challenge in the field due to the lack of non-invasive techniques which can be tuned at the tissue level.

## Conflicts of interest

There are no conflicts to declare.

## Supplementary Material
